# SARS-CoV-2 Delta Variant in Jingmen City, Hubei Province, China, 2021: Children Susceptible and Vaccination Breakthrough Infection

**DOI:** 10.3389/fmicb.2022.856757

**Published:** 2022-04-13

**Authors:** Dan Li, Ai-e Li, Zhu-qing Li, Yu Bao, Tian Liu, Xiang-Rong Qin, Xue-jie Yu

**Affiliations:** ^1^State Key Laboratory of Virology, School of Public Health, Wuhan University, Wuhan, China; ^2^Jingmen Municipal Health Commission, Jingmen, China; ^3^Jingmen Center for Disease Control and Prevention, Jingmen, China; ^4^Jingzhou Center for Disease Control and Prevention, Jingzhou, China; ^5^Department of Clinical Laboratory, The Second Hospital of Shandong University, Jinan, China

**Keywords:** SARS-CoV-2, delta variant (B.1.617.2), children, breakthrough infection, household transmission

## Abstract

**Background:**

The delta variant (B.1.617.2) of SARS-CoV-2 was the dominant viral strain causing COVID-19 in China, 2021. We reported a SARS-CoV-2 delta variant outbreak in Jingmen City, Hubei Province, China.

**Methods:**

The data of epidemiological, clinical, laboratorial, and vaccination of COVID-19 cases were collected through field investigation and analyzed.

**Results:**

During the outbreak from 4 to 20 August 2021, 58 cases of the SARS-CoV-2 delta variant (B.1.617.2) were identified with 15 (25.9%) asymptomatic and 43 (74.1%) symptomatic (mild and moderate) patients. The mean serial interval was 2.6 days (standard deviation: 2.0, 95% CI: 1.9–3.6). The median age of the patients was 39 years (ranging from 1 to 60 years) with the high proportion in children (19.0%). The secondary attack rate was 9.8% higher from parents to their children (<18 years) (46.2%, 95% CI: 14.8–77.5%) than that between spouses (36.4%, 95% CI: 14.5–58.2%), but no significant difference was observed (*p* > 0.05). Approximately half (27; 46.6%) of cases were vaccine breakthrough infections. In vaccine breakthrough cases (fully vaccinated), viral loads decreased 1.9–3.4-folds (*p* < 0.05), duration of viral shedding shortened 5 days (*p* < 0.05), and the risk of becoming symptomatic from asymptomatic decreased 33% (95% CI: 5–53%) (aged ≥12 years) than those in unvaccinated infections.

**Conclusions:**

Children are highly susceptible to the SARS-CoV-2 delta variant in the COVID-19 outbreak in Jingmen City in 2021. Inactivated vaccine derived from wide-type strain can effectively reduce the viral load, duration of viral shedding, and clinical severity in vaccine breakthrough cases. Our study indicates that protective measures that include full vaccination against COVID-19, especially in children, should be strengthened.

## Introduction

Since the pandemic of coronavirus disease 2019 (COVID-19) was reported in Wuhan in December 2019 (Li Q. et al., 2020), 416 million COVID-19 confirmed cases and over 5 million deaths were reported globally as of 17 February 2022 [WHO Coronavirus (COVID-19) Dashboard, 2022]. Currently, pediatric cases are on the rise composing 18.9% of all COVID-19 cases with nearly 7 million additional cases since the first week of September 2021 in the US (Children and COVID-19: State-Level Data Report, 2022). According to the definition of the World Health Organization (WHO), SARS-CoV-2 variants of concern have signature substitutions in key amino acids of the immunodominant spike protein which can affect virus characteristics with one or more of the following changes at a degree of global public health significance: increased transmissibility or detrimental change in COVID-19 epidemiology; increase in virulence or change in clinical disease presentation; and decrease in the effectiveness of public health and social measures or available diagnostics, vaccines, and therapeutics (Tracking SARS-CoV-2 Variants, 2022). Several SARS-CoV-2 variants of concern have emerged in the past 2 years including the alpha variant (B.1.1.7), beta variant (B.1.351), gamma variant (P.1), delta variant (B.617.2), lambda variant (C.37), and omicron variant (B.1.1.529) (Faria et al., 2021; Tegally et al., 2021; Vaidyanathan, 2021; Volz et al., 2021; Tracking SARS-CoV-2 Variants, 2022). The delta variant was first identified in October 2020 in India and rapidly spread to more than 200 countries, areas, or territories worldwide (Vaidyanathan, 2021; World Health Organization, 2021; Tracking of Variants, 2022; Tracking SARS-CoV-2 Variants, 2022). Based on the data from India and the UK, the delta variant was about 50–60% more transmissible than the alpha variant (Dhar et al., 2021; Public Health England, 2021).

In China, the first local transmission caused by the SARS-CoV-2 delta variant emerged in Guangzhou City in May 2021 (Zhang et al., 2021). Since then, the SARS-CoV-2 delta variant outbreaks have been reported in several cities and most of them infected <100 persons. However, there have been few publications related to SARS-CoV-2 delta variant outbreak in China, especially Hubei Province which was the epicenter of the first wave of the COVID-19 pandemic. Jingmen City in the middle of Hubei Province, a prefectural-level municipality with a population of 2.9 million, experienced the first wave of the COVID-19 pandemic with 928 confirmed cases between January and March 2020. The second COVID-19 outbreak caused by the delta variant occurred in August 2021. This outbreak gave us an opportunity to systematically analyze the characteristics of epidemiology, transmission dynamic, household transmission, and vaccination in the outbreak caused by the SARS-CoV-2 delta variant in Jingmen City, China.

## Methods

### Study Design and Data Sources

A retrospective observational study was conducted to explore the epidemiological, clinical, laboratorial, and effectiveness of vaccination in the SARS-CoV-2 delta variant outbreak in Jingmen City, Hubei Province, China, in August 2021. The relevant data were obtained by filed investigation and from official information system.

### Case Definition

The definitions of confirmed case, asymptomatic infection, close contact, and disease severity (mild, moderate, and severe) were all defined according to the China's national guidelines for the prevention and control of COVID-19 (the eighth version) and the guidelines for the diagnosis and treatment of COVID-19 (the eighth revised version) as described in Table 1 (National Health Commission of the People's Republic of China, 2021a,b). A COVID-19 vaccine breakthrough case was defined as a fully vaccinated case who received two doses of vaccine ≥14 days before the last exposure to a person with COVID-19.

**Table 1:** Case definition in the SARS-CoV-2 delta variant outbreak in Jingmen City, Hubei Province, China in August 2021.

**Terms**	**Definition**
Confirmed case	An individual who had positive PCR result of SARS-CoV-2 and COVID-19 related clinical symptoms (National Health Commission of the People's Republic of China, 2021a).
Asymptomatic infection	An individual who had positive PCR result of SARS-CoV-2 without clinical symptom (National Health Commission of the People's Republic of China, 2021a).
Close contact	An individual who was in contact without effective protection with someone with COVID-19 starting 4 days before the infected person developed symptoms or the sampling date if they did not have symptoms such as living, eating, working, traveling, and attending social activities (National Health Commission of the People's Republic of China, 2021a).
Mild COVID-19 case	A confirmed case with mild clinical symptoms, but without signs of pneumonia on chest imaging (National Health Commission of the People's Republic of China, 2021b).
Moderate COVID-19 case	A confirmed case with fever or respiratory symptoms, and imaging manifestations of pneumonia (National Health Commission of the People's Republic of China, 2021b).
Severe COVID-19 adult case	A confirmed case who met at least one of the following criteria: respiratory rate ≥30 times/min; oxygenation index ≤300 mmHg; resting oxygen saturation ≤93%; clinical symptoms were progressively aggravated and the lesion on lung had significantly progressed over 50% on chest imaging within 24–48 h (National Health Commission of the People's Republic of China, 2021b).
Severe COVID-19 child case	A confirmed case who met at least one of the following criteria: persistent high fever for more than 3 days; shortness of breath excluded the effects of fever and crying (respiratory rate in different ages: <2 months: ≥60 times/min; 2–12 months: ≥50 times/min; 1–5 years: ≥40 times /min;>5 years: ≥30 times/min); resting oxygen saturation ≤93%; assisted breathing; lethargy and convulsions; refusing food or feeding difficulties and dehydration (National Health Commission of the People's Republic of China, 2021b).

### Transmission Dynamic

The serial interval is the time between the onset of illness in the primary case and secondary case. The basic reproductive number (*R*_0_) is defined as the expected number of additional cases that one case will generate without intervention and control measures. The effective reproduction number (*R*_*t*_) is an indicator of real-time transmissibility of disease, which is often used to evaluate intervention and control measures. The incubation period is defined as the period of time between infection and illness onset.

### Household Transmission

A household was defined as two or more individuals living in a family in this study. Household contacts were defined as the individuals living in the same family for at least 1 day or night with the household index starting from 4 days before the onset or sampling of the index case (Li et al., 2021). For asymptomatic infections, onset was defined as the date of specimen collection for the first positive SARS-CoV-2 reverse transcription polymerase chain reaction (RT-PCR). A household secondary attack rate (SAR) was defined as the number of secondary symptomatic cases and asymptomatic cases in the household within 14 days after the index case was identified divided by the total number of contacts in the household.

### Effect of Vaccination

The COVID-19 vaccination history of each patient was obtained from the electronic health information system and field investigation. The vaccines used in Jingmen City were inactivated virions of SARS-CoV-2 wide-type strain and produced by Beijing Institute of Biological Products Co., Ltd, Wuhan Institute of Biological Products Co., Ltd, and Sinovac Life Science Co., Ltd.

The effect of inactivated SARS-CoV-2 vaccines against the delta variant was evaluated by comparing the clinical severity, RT-PCR cycle threshold (Ct) value, and duration of viral shedding of vaccinated patients with breakthrough infection or partially vaccinated to the unvaccinated. Asymptomatic case was considered as effective vaccination when evaluating the impact of the vaccine on clinical symptoms. Vaccine status was categorized as the unvaccinated, partly vaccinated, and fully vaccinated based on the fact that it takes 14 days to develop an effective protection against SARS-CoV-2 after vaccination (National Health Commission of the People's Republic of China, 2021b). The unvaccinated individuals were either not vaccinated before the last exposure to a person with COVD-19 or received one dose of vaccine <14 days before the last exposure. Partially vaccinated referred to individuals who received one dose of vaccine ≥14 days or received two doses of vaccine <14 days before the last exposure. Fully vaccinated referred to individuals who received two doses of vaccine ≥14 days before the last exposure.

### Laboratory Testing

The SARS-CoV-2 testing was performed according to the national guideline (National Health Commission of the People's Republic of China, 2021a). Nasopharyngeal swab samples were obtained from individuals, and RNA was extracted and tested by real-time RT-PCR with SARS-CoV-2-specific primers and probes aimed at the gene of the open reading frame (ORF) and nucleocapsid (N) protein. Cycle threshold (Ct) value <37 was defined as positive, and persons with the Ct value between 37 and 40 were resampled and tested to confirm. Viral load was estimated by the Ct value of real-time RT-PCR. A difference of 1 Ct unit was approximately equal to a factor of 2 in the number of virus per sample (Levine-Tiefenbrun et al., 2021). High-throughput sequencing was performed for SARS-CoV-2-positive specimens using MiniSeq high-output kit and Misq v2 Reagent Kit (Illumina, US) by the Hubei Provincial Center for Disease Control and Prevention. The whole genome sequence was compared with the nucleotide sequences in the GenBank and GISAID database.

### Statistical Analysis

The analysis of the serial interval, *R*_0_, *R*_*t*_, and incubation period, was performed with R software 4.0.5. The package of “*R*_0_” and “*EpiEstim*” was used for the estimation of *R*_0_ and *R*_*t*_, respectively. Categorical variables were compared with the chi-square test or the Fisher's exact test, and continuous variables were compared with the Mann–Whitney *U*-test using SPSS 25.0 as appropriate. Two-sided *p* < 0.05 and 95% confidence intervals (CIs) not including 1 value were considered as statistically significant.

### Ethics Approval and Consent

Institutional review board approval was waived as a part of a public health outbreak investigation in China. The data for analysis in our study were anonymous.

## Results

### Outbreak Description

**Figure 1:**
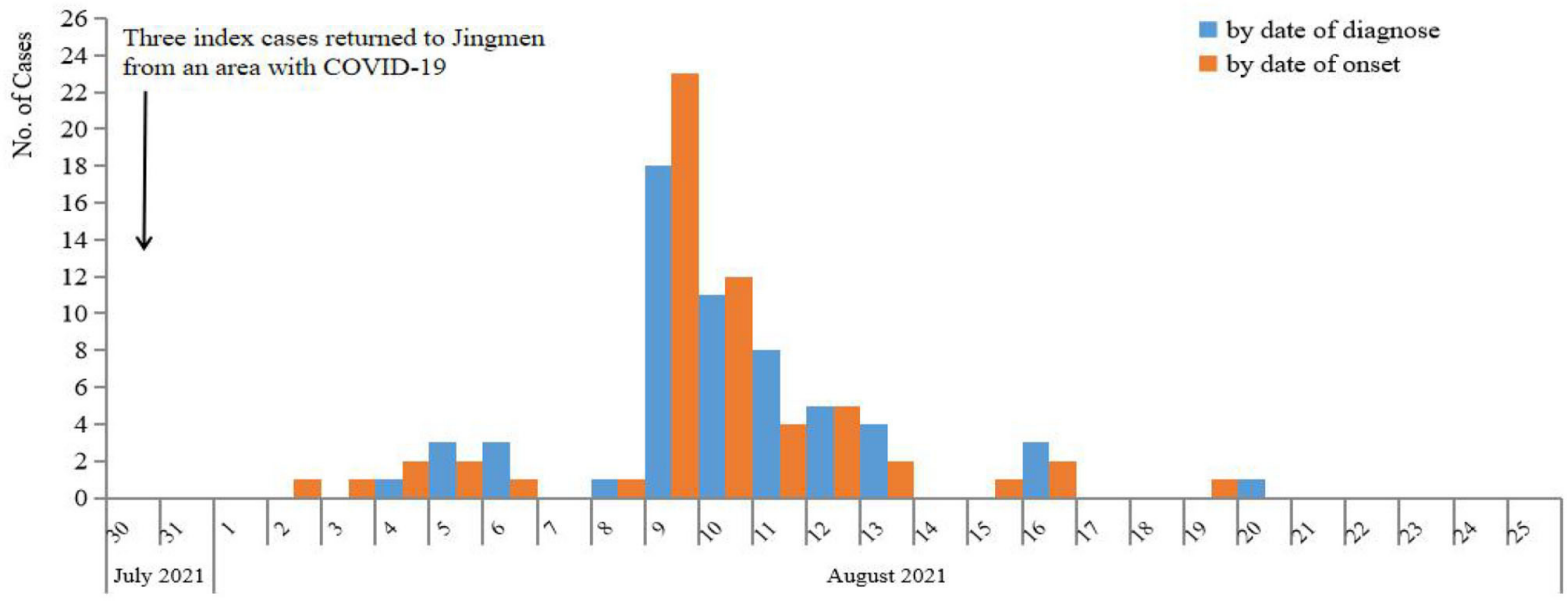
Epidemic curve of COVID-19 cases in Jingmen City, Hubei Province, China in August, 2021.

On 4 August 2021, a 33-year-old male who had illness onset 2 days ago was diagnosed as COVID-19 in Jingmen City, Hubei Province, China. The patient was a migrant worker and had worked in a construction filed in an area with COVID-19 before return to Jingmen City 5 days ago. After he was confirmed to be infected with SARS-CoV-2, another three co-workers who returned to Jinmen City together with the index patient were also tested for SARS-CoV-2 and two of his co-workers were also SARS-CoV-2-positive. After discovering the three patients, totally 4,436 close contacts were identified in Jingmen City, and all of them were tested for SARS-CoV-2 with RT-PCR from 4 to 20 August 2021. The PCR results showed that another 55 individuals to be SARS-CoV-2-positive. In total, 58 cases were identified in this COVID-19 epidemic in Jingmen City (Figure 1). DNA sequencing showed that the SARS-CoV-2 strains from the cases were delta variant. The median age of the cases was 39 years old ranging from 1 to 60 with 11 (19.0%) children (≤18 years), and the male and female ratio was 1.9:1 (38 men and 20 women). According to the clinical severity, 25.9% (15/58) of patients were asymptomatic, and 74.1% (43/58) of patients had clinical symptoms with 41.3% (24/58) mild and 32.8% (19/58) moderate. The asymptomatic proportion was 27.3% (3/11) for children and 25.5% (12/47) for adults (Fisher's exact: *p* > 0.05). The mean interval time between illness onset and diagnosis was 2 days (95% CI: 1.7–2.4). The median Ct value of the cases for the first real-time RT-PCR result was 26.3 (inter quartile range, IQR: 22.7–31.0) for the N gene and 27.35 (IQR: 22.0–32.23) for the ORF gene (Table 2).

**Table 2:** Epidemiological characteristics of the SARS-CoV-2 delta variant outbreak in Jingmen City, Hubei Province, China in August 2021.

**Characteristic**	**Delta variant positive persons (*N* = 58)**
	*n* (%)^*^
Age group, years	39 (1–60)
**Median age (range), years**	
≤18	11 (19.0)
19–29	5 (8.6)
30–39	15 (25.9)
40–49	11 (19.0)
50–59	14 (24.1)
≥60	2 (3.4)
**Gender**	
Female	20 (34.5)
Male	38 (65.5)
**Occupation**	
Worker	32 (55.2)
Preschool children^#^	8 (13.8)
Student	3 (5.2)
Teacher	1 (1.7)
Others	15 (25.9)
**District**	
A District where the index cases located	51 (87.9)
B District near A District	7 (12.1)
**Exposure history**	
Returned from an endemic area	3 (5.2)
Household contact	17 (29.3)
Social contact	38 (65.5)
**Clinical severity**	
Asymptomatic	15 (25.9)
Mild	24 (41.4)
Moderate	19 (32.8)
Severe and Critical	0 (0)
**Vaccination status**	
Unvaccinated	23 (39.7)
Partial vaccinated	8 (13.8)
Fully vaccinated	27 (46.6)
**Median Ct value of RT-PCR (IQR)**	
*N* gene	26.7 (22.9–31.0)
ORF1ab gene	27.4 (22.0–32.2)

* *n (%), number (percentage) unless otherwise specified; Ct, cycle threshold; IQR, inter quartile range*.

# *Preschool children, the children who did not attend kindergarten or school*.

### Transmission Dynamics

**Figure 2:**
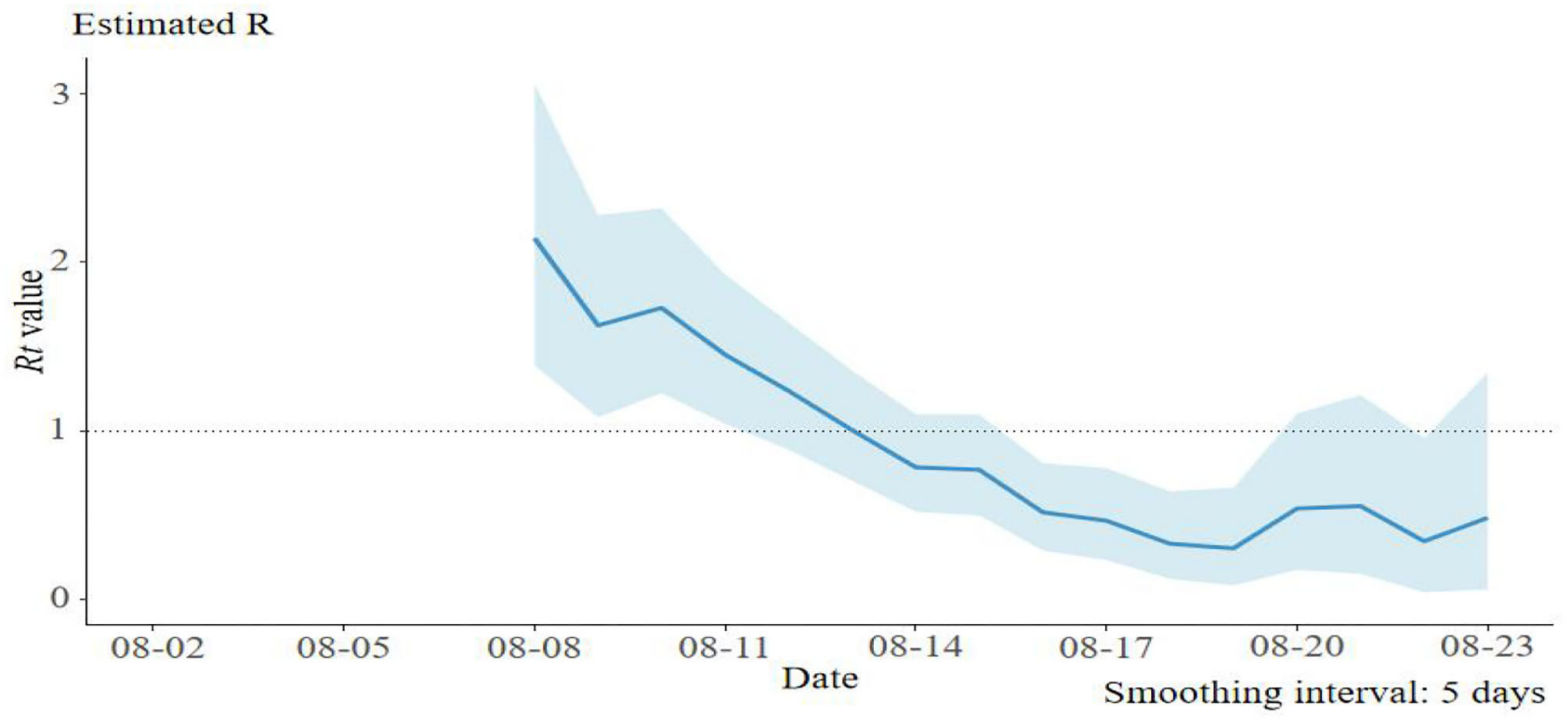
The effective reproduction number (*R*_*t*_) of the SARS-CoV-2 delta variant outbreak in Jingmen City, Hubei Province, China in August 2021.

Based on the epidemiological data of 58 infector–infectee pairs, the transmissibility parameters were calculated. The mean serial interval was 2.6 days (standard deviation: 2.0, 95% CI: 1.9–3.6) and mean incubation period was 4.0 days (95% CI: 2.0–4.8). The evaluated *R*_0_was 2.8 (95% CI: 1.6–4.8) using the maximum likelihood method (the coefficient of determination *R*^2^ = 0.96). Then, 9 days after the initial case was reported, the effective reproduction number (*R*_*t*_) decreased to 1 on 13 August, and fluctuated close to 1 from 13 to 20 August. No new case was reported since 20 August (Figure 2).

### Close Contacts Tracing

**Figure 3:**
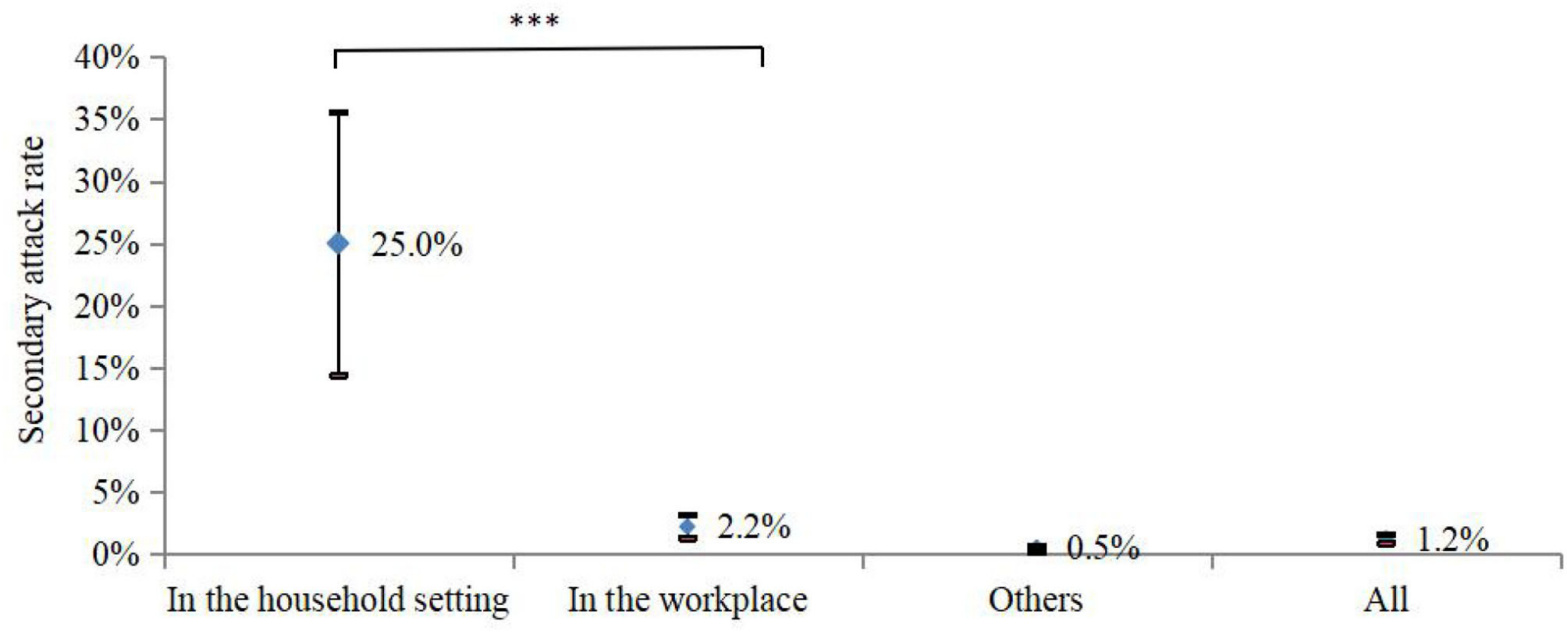
SAR occurred in the different settings in Jingmen City, Hubei Province, China in August 2021. ***p < 0.001.

In total, 4,436 close contacts were traced and quarantined, and 55 (1.2%, 95% CI: 0.9–1.6%) were found to be secondarily infected. The highest SAR occurred in the household setting (17/68, 25%, 95% CI: 14.4–35.6%), followed by 2.2% (23/1,025, 95% CI: 1.3–3.2%) in the workplace, and 0.5% (15/3,343, 95% CI: 0.2–0.7%) in other places, with significant differences (*p* < 0.05) (Figure 3).

### Household Transmission

**Figure 4:**
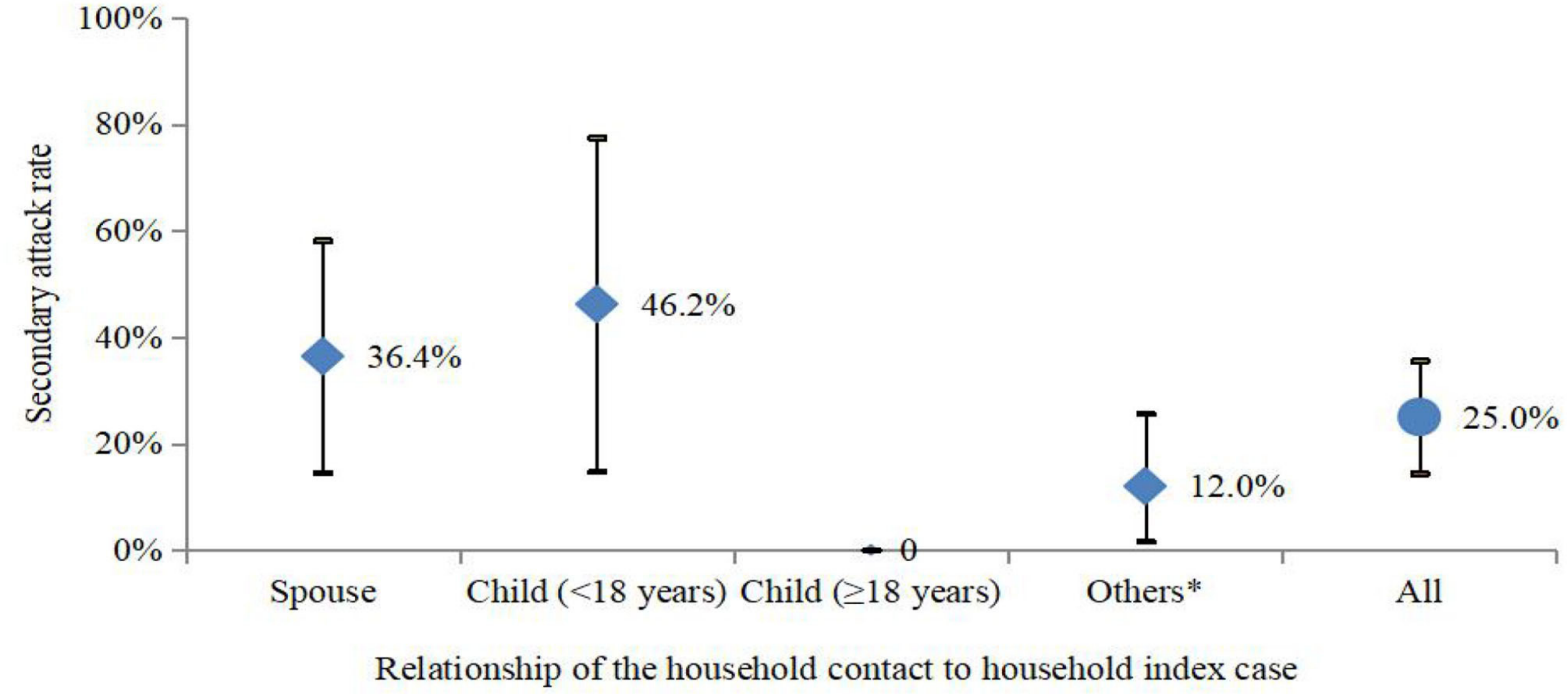
SAR (95% CI) in households in the SARS-CoV-2 delta variant outbreak in Jingmen City, Hubei Province, China in August 2021. *Others included parents, siblings, and grandparents.

Among all 58 COVID-19 cases, 44 cases belonged to 27 households and 14 cases lived alone or in working dormitories. In 27 households with COVID-19 cases, there were 68 household contacts with the median number of persons per household of three, which ranged from 2 to 7. For the 27 households with COVID-19, 11 households (39.3%) had 17 secondary cases. The SAR was 25.0% (17/68) (95% CI: 14.4–35.6%) among the household contacts. Although the SAR was 9.8% higher between parents and children (<18 years) (46.2%, 95% CI: 14.8–77.5%) than that between spouses (36.4%, 95% CI: 14.5–58.2%), no statistical difference was observed (*p* > 0.05) (Figure 4). Among the 11 households with secondary transmission, the SAR was 60.7% (17/28, 95% CI: 41.4–80.0%), and 3 (27.3%) out of 11 households had a 100% SAR. The average number of secondarily infected individuals was 2, which ranged from 2 to 6, and the median serial interval was 2.0 days (standard deviation: 2.2, 95% CI: 1.5–4.0).

The median Ct values for the N gene and ORF1ab gene were 23.1 (21.7–26.4) and 23.2 (21.1–27.9) in households with secondary transmission and 27.1 (22.4–29.1) and 29.1 (23.0–31.2) in household without secondary transmission, which represented an increase of 4.0–5.9-folds in viral load in household indexes with secondary transmission compared to those without secondary transmission (*p* < 0.05), which indicated a high viral load might contribute to secondary transmission. There was no statistically difference in the age, gender, and clinical severity between the household indexes with secondary transmission and those without secondary transmission (*p* > 0.05) (Table 3).

**Table 3:** Characteristics of household indexes in the SARS-CoV-2 delta variant outbreak in Jingmen City, Hubei Province, China in August 2021.

**Characteristics of household indexes**	**With secondary transmission (*n* = 11)**	**Without secondary transmission (*n* = 16)**	*p*-value^*^
Median age (rang), years	42 (35–50)	43 (30–51)	>0.05
**Gender**			
Female	1	5	>0.05
Male	10	11	
**Clinical severity**			
Symptomatic cases	8	10	>0.05
Asymptomatic infections	3	6	
**RT-PCR median Ct value (IQR)**			
*N* gene	23.1 (21.7–26.4)	27.1 (22.4–29.1)	<0.05
ORF1ab gene	23.2 (21.1–27.9)	29.1 (23.0–31.2)	<0.05

*Ct, cycle threshold; IQR, inter quartile range*.

* *Categorical variables (gender and clinical severity) were compared by the Fisher's exact test, and continuous variables (median age and RT-PCR median Ct value) were compared by Mann–Whitney U-test*.

### The Effect of Vaccination

Among the 58 COVID-19 cases, 27 (46.6%) were vaccine breakthrough cases (fully vaccinated with inactivated virion vaccine of wide-type strain). The first-time PCR results showed that the median Ct values of both N gene and ORF1ab gene in vaccine breakthrough cases were higher than that in unvaccinated cases (Table 4), which represents a 1.9–3.4-fold decrease of viral load in vaccine breakthrough cases compared to the unvaccinated cases (*p* < 0.05), while no statistical difference was observed in partially vaccinated cases compared to the unvaccinated cases (*p* > 0.05). The duration of viral shedding was 5 days shorter in vaccine breakthrough cases (median: 19 days; IQR: 15–23 days) than that in unvaccinated cases (median: 24 days; IQR: 18–30 days) (*p* < 0.05) (Table 4). The risk of progressing from asymptomatic to symptomatic decreased 33% (95% CI: 5–53%) in fully vaccinated patients than that in unvaccinated patients aged ≥12 years (Table 5).

**Table 4:** The viral load of the SARS-CoV-2 delta variant infected patients in different vaccination status in Jingmen City, Hubei Province, China in August 2021.

**Characteristics**	**Unvaccinated (IQR)**	**Fully vaccinated (IQR)**	^*^p-value (fully vaccinated vs. unvaccinated)	**Partially vaccinated (IQR)**	^*^p-value (partially vaccinated vs. unvaccinated)
RT-PCR median Ct value of N gene	25.1 (23.0–32.7)	27.0 (21.1–29.8)	<0.05	28.0 (25.2–30.2)	>0.05
RT-PCR median Ct value of ORF lab gene	25 (23.0–32.6)	28.4 (21.0–31.1)	<0.05	28.9 (25.5–31.6)	>0.05
Median duration of viral shedding, days	24 (18–30)	19 (15–23)	<0.05	19 (14–38)	<0.05

*QR, inter quartile range*.

* *Continuous variables were compared by Mann–Whitney U-test*.

**Table 5:** Clinical severity of delta variant infected patients in different vaccination status in Jingmen City, Hubei Province in August 2021.

**Outcome**	**Unvaccinated**	**Partially vaccinated**			**Fully vaccinated**		
	***n***	***n***	**RR (95% CI)**	^*^Reduced risk % (95% CI)	***n***	**RR (95% CI)**	^*^Reduced risk % (95% CI)
**The COVID-19 infection (*****N*** **=** **58)**							
Symptomatic	19	7	1.35 (0.21–8.68)	−35 (−768 to 79)	17	0.66 (0.41–1.07)	34 (−7 to 59)
Moderate	8	5	1.59 (0.50–5.09)	−59 (−409 to 50)	6	0.87 (0.53–1.43)	13 (−43 to 47)
Mild	11	2	0.83 (0.22–3.18)	17 (−218 to 78)	11	0.78 (0.49–1.24)	22 (−24 to 51)
Asymptomatic	4	1	Ref.		10	Ref.	–
**Age≥12 years** **(*****N*** **=** **47)**							
Symptomatic	11	7	0.78 (0.17–3.49)	22 (−249 to 83)	17	0.67 (0.47–0.95)	33 (5–53)
Moderate	5	5	1 (0.22–4.56)	0 (−356 to 78)	6	0.6 (0.34–1.062)	40 (−6 to 66)
Mild	6	2	0.5 (0.08–3.13)	50 (−213 to 92)	11	0.71 (0.48–1.06)	29 (−6 to 52)
Asymptomatic	1	1	Ref.	–	10	Ref.	Ref.

* *Reduced risk compared with the unvaccinated; Ct, cycle threshold; IQR, inter quartile range; RR, risk ratio; 95% CI, 95% confidence interval; –, not available*.

## Discussion

The SARS-CoV-2 delta variant outbreak in Jingmen City in August 2021 was eliminated in 17 days with 58 cases and the proportion of infected children was relatively high (19%) in this outbreak. In the first wave of COVID-19 pandemic caused by wide-type SARS-CoV-2 in Jingmen City in 2020, the proportion of infected children was only 0.5% (5/928). The previous study showed that children appeared to be less infected by the wide-type strain of SARS-CoV-2 (infection rate varied from 0.9–7.9%) than adults based on a large scale COVID-19-related epidemiological studies (Mehta et al., 2020). A meta-analysis based on 213 published literatures of household transmission showed that the SAR of other strains of SARS-CoV-2 in pediatric household contacts was lower than in adult household contacts (RR, 0.62; 95% CI, 0.42–0.91) (Zhu et al., 2021). We found that all pediatric cases were between ages 1 and 10 years in this outbreak and did not get vaccinated. Vaccination might be a confounding factor to affect the susceptibility in children under 12 years to delta variant. However, there was no vaccine available during wide-type strain epidemic in China. Children under 12 years were not vaccinated both in the previous wide-type strain epidemic and this delta variant outbreak in Jingmen City. Therefore, it could be ruled out that the vaccine affects the susceptibility of children to the wide-type strain outbreaks and this outbreak. Children are more susceptible to SARS-CoV-2 delta variant in Jingmen City in 2021 than wide-type strain in 2019–2020, which could be caused by different exposure opportunity and/or viral type.

The SAR between parents and children (<18 years) was much higher (9.8%) than that between spouses in our study. However, no statistical difference was observed (*p* > 0.05), which was most likely caused by the small sample size. The higher SAR in children than parents in the household could be influenced by the mode of interactions between the adults and children (as in preparation of food, for instance), or more greatly associated with a particular type of interaction, or vaccination status and not an inherent increased susceptibility of children. Whether children are more susceptible than adults to SARS-CoV-2 delta variant needs to be further investigated.

As of 4 February 2022, a total of 8.6 billion doses of COVID-19 vaccine have been administered globally including 3.0 billion in China (WHO, 2022). COVID-19 vaccine breakthrough infections with varied proportion have been observed (Abu-Raddad et al., 2021; Bergwerk et al., 2021). In this study, we systematically evaluated the effect of vaccination derived from the wide-type strain in viral loads, duration of viral shedding, and clinical severity in the outbreak of SARS-CoV-2 delta variant in Jingmen City in 2021. Our findings showed that full vaccination with inactivated COVID-19 vaccines derived from the wide-type strain had partial cross-protection against the SARS-CoV-2 delta variant (B.1.617.2). In vaccine breakthrough cases, the SARS-CoV-2 delta variant viral load in the first positive PCR test and duration of viral shedding decreased 1.9–3.4-folds and shortened 5 days, respectively, compared with those in unvaccinated patients. The previous study in Israel (*n* = 3,776) showed a decrease of 2.8–4.5-folds in SARS-CoV-2 viral load in BNT162b2 messenger RNA vaccine breakthrough patients compared with those of the unvaccinated (Levine-Tiefenbrun et al., 2021). Our study showed that inactivated SARS-CoV-2 vaccine derived from the wide-type strain also had the similar function in reducing the viral load in the vaccine breakthrough infections of SARS-CoV-2 delta variant. A study in the US between January and August 2021 showed that symptomatic delta variant breakthrough cases had longer duration of viral shedding compared with non-delta variants (Siedner et al., 2022). However, to our knowledge, few published studies explored the difference of the viral shedding duration between the vaccine breakthrough infections and the unvaccinated for the individuals who both infected with SARS-CoV-2 delta variant. This study added the knowledge to this aspect. Vaccine breakthrough patients aged ≥12 years had a 33% reduced risk in progressing from asymptomatic to symptomatic compared with that in unvaccinated cases in this outbreak. A SARS-CoV-2 delta variant outbreak that occurred in Guangzhou City, China, in May and June 2021 showed that the function of inactive full vaccination was 59.0% vaccination effectiveness against infection, 70.2% against moderate COVID-19, 100% against severe by case-control study, 51.8% against infection, 60.4% against symptomatic infection, 78.4% against pneumonia, and 100% against severe or critical illness by retrospective cohort study (Li et al., 2021; Kang et al., 2022). A previous study in the UK showed that the BNT162b2 and ChAdOx1 nCoV-19 vaccines were 88.0 and 67.0% effectiveness against the SARS-COV-2 delta variant (Lopez Bernal et al., 2021).

In our study, the mean serial interval was 2.6 days, which was similar to what was observed in the SARS-CoV-2 delta variant outbreak in Guangdong Province (2.3 days), but significantly shorter than what was observed from the wide-type strain of SARS-CoV-2 outbreak in Wuhan City (early estimation: 7.5 days) (Li Q. et al., 2020), Jingzhou City (5.8 days) (Liu et al., 2020), Hubei Province (4.0 days) (Du et al., 2020), and Hunan Province (5.5 days) in 2020 (Hu et al., 2021). The shorter serial interval of SARS-CoV-2 delta variant implies control measures such as contact tracing and quarantining which should be conducted as soon as possible to stop further transmission. The effective reproduction number (*R*_*t*_) was an indicator of real-time transmissibility of disease and the effect of interventions. In this epidemic, *R*_*t*_decreased to about 1, 9 days after the first case was reported, which was similar to the delta variant outbreak in Guangzhou City (Zhang et al., 2021). A series of rigorous intervention measures were used to contain this outbreak, which includes active case finding, rapid contacts tracing and quarantining, three times of nucleic acid test for the whole population in the city, lockdown of affected area, community management, and travel restrictions. The quick decline of *R*_*t*_indicated that the control measures worked well.

High transmissibility in households was another feature in this outbreak. The study of household SARs caused by the SARS-CoV-2 delta variant (B.1.617.2) was limited (Dougherty et al., 2021). In the previous studies of epidemics caused by the wide-type of SARS-CoV-2 or other related variants, household SARs varied from 3 to 32% in China and the US (Jing et al., 2020; Lewis et al., 2020; Li W. et al., 2020; Wu et al., 2020; Cerami et al., 2021). Compared with outbreaks caused by wide-type strain of SARS-CoV-2, the overall household SAR in this outbreak (25%) was higher than that in Guangzhou City, China (12.4–17.1%) and lower than that in the US households (29–32%) and Zhuhai City, China (32%) (Jing et al., 2020; Lewis et al., 2020; Wu et al., 2020; Cerami et al., 2021). However, high SAR (60.7%) was observed among households with secondary transmission including 100% SAR in 3 households in this outbreak, which was similar to SARS-CoV-2 delta variant (B.1.617.2) outbreak in Oklahoma, US (SARs among households with secondary transmission ranged from 80 to 100%) (Dougherty et al., 2021). The viral load in index patients with household secondary transmission was much higher (4.0–5.9-folds) than those index patients without household secondary transmission. Factors such as household contacts without protective measures cohabitation with the index case and household crowding may increase the risk of secondary infection (Lewis et al., 2020; Wu et al., 2020; Cerami et al., 2021). In our study, although the primary infections were sent to the designated hospitals as soon as possible, precautionary practices such as mask use and increased social distance basically were not observed in households, which probably increased the odds of household transmission (Wu et al., 2020).

Our study has several limitations. First, recall bias could not be fully avoided in the investigation of cases and their contacts despite the in-depth investigation. Second, the sample size in this outbreak is small, which may affect the statistical test. Third, the valuation of vaccination effectiveness by respective cohort study, case–control, or subgroup analyses was limited by insufficient data such as few cases, no severe and critical case, and unavailable data for the vaccination status of all close contacts in this study. Fourth, although the hospitalization is an important index to evaluate the effectiveness of vaccination, we could not use it because all individuals infected with SARS-CoV-2 were hospitalized even if asymptomatic in China.

In conclusion, our findings showed that children are highly susceptible to the SARS-CoV-2 delta variant (B.1.617.2), and the transmission could easily happen from parents to their children. Inactivated COVID-19 vaccine derived from wide-type strain of SARS-CoV-2 showed protection against the SARS-CoV-2 delta variant in reducing viral load, duration of viral shedding, and disease severity in vaccine breakthrough cases compared with those in unvaccinated cases. Protective measures against COVID-19 among children should be strengthened, and increased full vaccination efforts should be taken not only in adults, but also in children to reduce the disease severity in individuals and prevent the spread of the virus in the community.

## Data Availability Statement

The original contributions presented in the study are included in the article/supplementary material, further inquiries can be directed to the corresponding author/s.

## Ethics Statement

Ethical review and approval was not required for the study on human participants in accordance with the local legislation and institutional requirements. Written informed consent from the participants' legal guardian/next of kin was not required to participate in this study in accordance with the national legislation and the institutional requirements.

## Author Contributions

DL contributed to field investigation, analysis, and manuscript drafting. A-eL contributed to study design and supervision. Z-qL and YB contributed to field investigation. TL contributed to field investigation and revised the manuscript. X-RQ contributed to supervision. X-jY contributed to study design, revised the manuscript, and supervised the study. All authors contributed to the article and approved the submitted version.

## Funding

This work was supported by the National Natural Science Funds of China (grant number: 81971939), the Natural Science Foundation of Shandong Province of China (ZR202102210339), and Jingmen Science and Technology Plan Project (grant number: 2020YFYB114).

## Conflict of Interest

The authors declare that the research was conducted in the absence of any commercial or financial relationships that could be construed as a potential conflict of interest.

## Publisher's Note

All claims expressed in this article are solely those of the authors and do not necessarily represent those of their affiliated organizations, or those of the publisher, the editors and the reviewers. Any product that may be evaluated in this article, or claim that may be made by its manufacturer, is not guaranteed or endorsed by the publisher.

## References

[B1] Abu-Raddad L. J. Chemaitelly H. Ayoub H. H. Yassine H. M. Benslimane F. M. Al Khatib H. A.. (2021). Association of prior SARS-CoV-2 infection with risk of Breakthrough infection following mRNA vaccination in Qatar. JAMA 326, 1930–1939. 10.1001/jama.2021.19623 34724027 PMC8561432

[B2] Bergwerk M. Gonen T. Lustig Y. Amit S. Lipsitch M. Cohen C.. (2021). Covid-19 breakthrough infections in vaccinated health care workers. N. Engl. J. Med. 385, 1474–1484. 10.1056/NEJMoa2109072 34320281 PMC8362591

[B3] Cerami C. Popkin-Hall Z. R. Rapp T. Tompkins K. Zhang H. Muller M. S.. (2021). Household transmission of SARS-CoV-2 in the United States: living density, viral load, and disproportionate impact on communities of color. Clin. Infect. Dis. 12, ciab701. 10.1093/cid/ciab701 34383889 PMC8436395

[B4] Children COVID-19: State-Level Data Report. (2022) Available online at: https://www.aap.org/en/pages/2019-novel-coronavirus-covid-19-infections/children-and-covid-19-state-level-data-report/ (accessed February 18 2022).

[B5] Dhar M. R. Radhakrishnan V. Ponnusamy K. Rakshit P. (2021). Genomic characterization and epidemiology of an emerging SARS-CoV-2 variant in Delhi, India. Science. 374, 995–999. 10.1126/science.abj9932 34648303 PMC7612010

[B6] Dougherty K. Mannell M. Naqvi O. Matson D. Stone J. (2021). SARS-CoV-2 B.1.617.2 (Delta) Variant COVID-19 outbreak associated with a gymnastics facility - Oklahoma. Morb. Mortal. Wkly. Rep. 70, 1004–1007. 10.15585/mmwr.mm7028e2 34264910 PMC8314708

[B7] Du Z. W. Xu X. K. Wu Y. Wang L. Cowling B. J. Meyers L. A. (2020). Serial interval of COVID-19 among publicly reported confirmed cases. Emerg. Infect. Dis. 26, 1341–1343. 10.3201/eid2606.200357 32191173 PMC7258488

[B8] Faria N. R. Mellan T. A. Whittaker C. Claro I. M. Candido D. D. S. Mishra S.. (2021). Genomics and epidemiology of the P.1 SARS-CoV-2 lineage in Manaus, Brazil. Science 372, 815–821. 10.1126/science.abh2644 33853970 PMC8139423

[B9] Hu S. Wang W. Wang Y. Litvinova M. Luo K. Ren L.. (2021). Infectivity, susceptibility, and risk factors associated with SARS-CoV-2 transmission under intensive contact tracing in Hunan, China. Nat. Commun. 12, 1533. 10.1038/s41467-021-21710-6 33750783 PMC7943579

[B10] Jing Q. L. Liu M. J. Zhang Z. B. Fang L. Q. Yuan J. Zhang A. R.. (2020). Household secondary attack rate of COVID-19 and associated determinants in Guangzhou, China: a retrospective cohort study. Lancet Infect. Dis. 20, 1141–1150. 10.1016/S1473-3099(20)30471-0 32562601 PMC7529929

[B11] Kang M. Yi Y. Li Y. Sun L. M. Deng A. P. Hu T.. (2022). Effectiveness of inactivated COVID-19 vaccines against illness caused by the b.1.617.2 (Delta) variant during an Outbreak in Guangdong, China. Ann. Intern. Med. M21–3509. 10.7326/M21-3509 35099990 PMC8819853

[B12] Levine-Tiefenbrun M. Yelin I. Katz R. Herzel E. Golan Z. Schreiber L.. (2021). Initial report of decreased SARS-CoV-2 viral load after inoculation with the BNT162b2 vaccine. Nat. Med. 27, 790–792. 10.1038/s41591-021-01316-7 33782619

[B13] Lewis N. M. Chu V. T. Ye D. Conners E. E. Gharpure R. Laws R. L.. (2020). Household transmission of SARS-CoV-2 in the United States. Clin. Infect. Dis. 73, 1805–1813. 10.1093/cid/ciaa1166 33185244 PMC7454394

[B14] Li Q. Guan X. Wu P. Wang X. Zhou L. Tong Y.. (2020a). Early transmission dynamics in Wuhan, China, of novel coronavirus-infected pneumonia. N. Engl. J. Med. 382, 1199–1207. 10.1056/NEJMoa2001316 31995857 PMC7121484

[B15] Li W. Zhang B. Lu J. Liu S. Chang Z. Peng C.. (2020b). Characteristics of household transmission of COVID-19. Clin. Infect. Dis. 71, 1943–1946. 10.1093/cid/ciaa450 32301964 PMC7184465

[B16] Li X. N. Huang Y. Wang W. Jing Q. L. Zhang C. H. Qin P. Z.. (2021). Effectiveness of inactivated SARS-CoV-2 vaccines against the Delta variant infection in Guangzhou: a test-negative case-control real-world study. Emerg. Microbes Infect. 10, 1751–1759. 10.1080/22221751.2021.1969291 34396940 PMC8425710

[B17] Liu T. Qi L. Yao M. Tian K. Lin M. Jiang H. (2020). Serial interval and reproductive number of COVID-19 among 116 infector-infectee Pairs — Jingzhou City, Hubei Province, China, 2020. China CDC Wkly. 2, 491–495. 10.46234/ccdcw2020.118 34594686 PMC8428449

[B18] Lopez Bernal J. Andrews N. Gower C. Gallagher E. Simmons R. Thelwall S.. (2021). Effectiveness of Covid-19 vaccines against the B.1.617.2 (Delta) variant. N. Engl. J. Med. 385, 585–594. 10.1056/NEJMoa2108891 34289274 PMC8314739

[B19] Mehta N. S. Mytton O. T. Mullins E. W. S. Fowler T. A. Falconer C. L. Murphy O. B.. (2020). SARS-CoV-2 (COVID-19): what do we know about children? A systematic review. Clin. Infect. Dis. 71, 2469–2479. 10.1093/cid/ciaa556 32392337 PMC7239259

[B20] National Health Commission of the People's Republic of China. (2021a). Guidelines for the Prevention and Control of COVID-19 (the eighth version). Available online at: http://www.nhc.gov.cn/jkj/s3577/202105/6f1e8ec6c4a540d99fafef52fc86d0f8.shtml (accessed September 1, 2021).

[B21] National Health Commission of the People's Republic of China. (2021b). Guidelines for the Diagnosis and Treatment of COVID-19 (the eighth version). Available online at: http://www.nhc.gov.cn/jkj/s3577/202105/6f1e8ec6c4a540d99fafef52fc86d0f8.shtml (accessed September 1, 2021).

[B22] Public Health England. (2021) SARS-CoV-2 Variants of Concern and Variants Under Investigation in England: Technical Briefing 13. Available online at: https://assets.publishing.service.gov.uk/government/uploads/system/uploads/attachment_data/file/990339/Variants_of_Concern_VOC_Technical_Briefing_13_England.pdf (accessed September 18 2021).

[B23] Siedner M. J. Boucau J. Gilbert R. F. Uddin R. Luu J. Haneuse S.. (2022). Duration of viral shedding and culture positivity with postvaccination SARS-CoV-2 delta variant infections. JCI Insight 7, e155483. 10.1172/jci.insight.155483 34871181 PMC8855795

[B24] Tegally H. Wilkinson E. Giovanetti M. Iranzadeh A. Fonseca V. Giandhari J.. (2021). Detection of a SARS-CoV-2 variant of concern in South Africa. Nature 592, 438–443. 10.1038/s41586-021-03402-9 33690265

[B25] Tracking of Variants. (2022) Available online at: https://www.gisaid.org/hcov19-variants/ (accessed February 18 2022).

[B26] Tracking SARS-CoV-2 Variants. (2022) Available online at: https://www.who.int/en/activities/tracking-SARS-CoV-2-variants/ (accessed February 18, 2022).

[B27] Vaidyanathan G. (2021). Coronavirus variants are spreading in India - what scientists know so far. Nature 593, 321–322. 10.1038/d41586-021-01274-7 33976409

[B28] Volz E. Mishra S. Chand M. Barrett J. C. Johnson R. Geidelberg L.. (2021). Assessing transmissibility of SARS-CoV-2 lineage B.1.1.7 in England. Nature 593, 266–269. 10.1038/s41586-021-03470-x 33767447

[B29] WHO Coronavirus (COVID-19) Dashboard. (2022) Available online at: https://covid19.who.int/ (accessed February 18, 2022).

[B30] World Health Organization. (2021) CORONAVIRUS disease (COVID-19) Weekly Epidemiological Update and Weekly Operational Update. Available online at: https://www.who.int/publications/m/item/weekly-epidemiological-update-on-covid-19-8-june-2021 (accessed September 18 2021).

[B31] Wu J. Huang Y. Tu C. Bi C. Chen Z. Luo L.. (2020). Household transmission of SARS-CoV-2, Zhuhai, China, 2020. Clin. Infect. Dis. 71, 2099–2108. 10.1093/cid/ciaa557 32392331 PMC7239243

[B32] Zhang M. Xiao J. Deng A. Zhang Y. Zhuang Y. Hu T.. (2021). Transmission dynamics of an outbreak of the COVID-19 delta variant B.1.617.2 — Guangdong Province, China. China CDC Weekly 3, 584–586. 10.46234/ccdcw2021.151 34594941 PMC8392962

[B33] Zhu Y. Bloxham C. J. Hulme K. D. Sinclair J. E. Tong Z. W. M. Steele L. E.. (2021). A meta-analysis on the role of children in severe acute respiratory syndrome coronavirus 2 in household transmission clusters. Clin. Infect. Dis. 72, e1146–e1153. 10.1093/cid/ciaa1825 33283240 PMC7799195

